# Data missingness in the Michigan NEMSIS (MI-EMSIS) dataset: a mixed-methods study

**DOI:** 10.1186/s12245-021-00343-y

**Published:** 2021-04-14

**Authors:** Mahshid Abir, Rekar K. Taymour, Jason E. Goldstick, Rosalie Malsberger, Jane Forman, Stuart Hammond, Kathy Wahl

**Affiliations:** 1 Department of Emergency Medicine, University of Michigan, Ann Arbor, MI USA; 2 Acute Care Research Unit, Institute for Healthcare Policy and Innovation, University of Michigan, Ann Arbor, MI USA; 3 RAND Corporation, Santa Monica, CA USA; 4 PRECISIONValue, Detroit, MI USA; 5 Mathematica, Boston, MA USA; 6 Veterans Affairs Ann Arbor Healthcare System, Ann Arbor, MI USA; 7 Michigan Department of Health and Human Services, Lansing, MI USA

**Keywords:** Emergency Medical Services, Quality assurance, Prehospital health care, Quality measure, Data, Big data, Data collection

## Abstract

**Objective:**

The study was done to evaluate levels of missing and invalid values in the Michigan (MI) National Emergency Medical Services Information System (NEMSIS) (MI-EMSIS) and explore possible causes to inform improvement in data reporting and prehospital care quality.

**Methods:**

We used a mixed-methods approach to study trends in data reporting. The proportion of missing or invalid values for 18 key reported variables in the MI-EMSIS (2010–2015) dataset was assessed overall, then stratified by EMS agency, software platform, and Medical Control Authorities (MCA)—regional EMS oversight entities in MI. We also conducted 4 focus groups and 10 key-informant interviews with EMS participants to understand the root causes of data missingness in MI-EMSIS.

**Results:**

Only five variables of the 18 studied exhibited less than 10% missingness, and there was apparent variation in the rate of missingness across all stratifying variables under study. No consistent trends over time regarding the levels of missing or invalid values from 2010 to 2015 were identified. Qualitative findings indicated possible causes for this missingness including data-mapping issues, unclear variable definitions, and lack of infrastructure or training for data collection.

**Conclusions:**

The adoption of electronic data collection in the prehospital setting can only support quality improvement if its entry is complete. The data suggest that there are many EMS agencies and MCAs with very high levels of missingness, and they do not appear to be improving over time, demonstrating a need for investment in efforts in improving data collection and reporting.

## Introduction

### Background

In 2015, the Institute of Medicine (IOM), now named the National Academy of Medicine (NAM), stated that emergency medical services (EMS) systems exhibit fragmentation and an absence of system-wide coordination and planning [1]. Effective EMS oversight requires reliable data, the need for which has been recognized as early as the 1970s [2], but it was not until 2002 that the National Highway Traffic Safety Administration (NHTSA) and the Health Resources and Services Administration (HRSA) sponsored a forum leading to the EMS Performance Measures Project which, in 2009, identified 35 measures for EMS systems [3]. These indicators, or quality measures, served as the basis of the National EMS Information System (NEMSIS)—a dataset assembled with the goal of storing data reported by all EMS agency types (e.g., non-transporting agencies, ground-transporting agencies, and both rotor and fixed-wing flight services) from every state in the nation for all levels of services (e.g., medical first response through Advanced Life Support) (Kevin Putnam, EMS State Data Manager) [4].

In Michigan, EMS system oversight in the state’s 83 counties is conducted by 60 Medical Control Authorities (MCAs), organizations designated by the Michigan Department of Health and Human Services (MDHHS) [5, 6]. MCA oversight entails supervision and coordination of local EMS where MCAs provide medical direction, educate clinicians, and implement and enforce local and statewide protocols [6–8]. Pursuant to state policy, MCAs are additionally responsible for establishing a professional standards review organization to evaluate and improve EMS agency operations based on current performance standards by establishing quality improvement protocol and programming for EMS agencies, collecting patient care and performance data from each agency within the MCA region, and providing data and performance metrics to the MDHHS [5, 7].

**Fig. 1:**
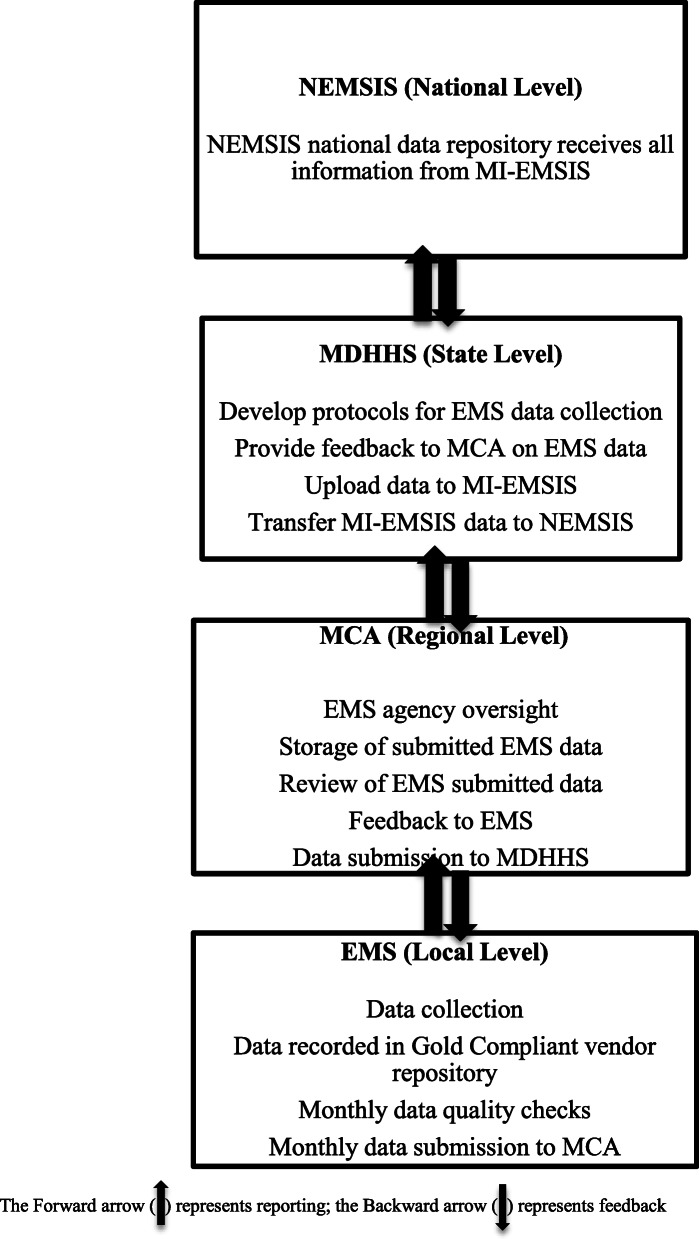
MI-EMSIS data flow and feedback process. The forward arrow (↑) represents reporting; the backward arrow (↓) represents feedback*. Figure adapted using* [6, 7]

NEMSIS is the most prominent prehospital quality reporting dataset and serves as a standardized national repository of prehospital EMS data [4]. Currently, over 90% of US states and territories have a NEMSIS compliant data system in place [4]. NEMSIS compliance is defined as the use of Gold Compliant data repositories, meaning vendor software is properly configured for sending and receiving properly formatted EMS data from agencies to the state, then on to NEMSIS (Kevin Putnam, EMS State Data Manager) [7–13]. To maintain NEMSIS compliance, the vendors used by EMS agencies for data collection and reporting must stay up to date with the most current versions of NEMSIS, updated to meet evolving needs for prehospital data collection and make consistent with other prehospital and emergency setting standards [13–15]. Michigan does not require the use of specific vendors by EMS agencies, the state only mandates vendor Gold Compliant. At the local (EMS) reporting level in Michigan, the NEMSIS-compliant vendor endorsed by MDHHS for uploading data to the state repository (MI-EMSIS) is ImageTrend, although many agencies report using third-party data repositories [6, 12–15]. Michigan maintains a list of 287 data elements from the 430 included in NEMSIS which agencies are required to report on [16–18]. Agency-reported data is collected and stored in either Imagetrend or third-party repositories and is then uploaded by MCAs into the statewide NEMSIS compliant system. In Michigan (MI), the NEMSIS-compliant statewide EMS information system is referred to as the Michigan EMS Information System (MI-EMSIS). Data stored in the NEMSIS system is accessible to all reporting agencies and to MCAs which have completed the necessary agreements for access to records which occurred within their geographic region by their agencies (Kevin Putnam, EMS State Data Manager). One significant challenge for EMS agency data reporting at the oversight level in Michigan is the difference in structure and operation of EMS systems from location to location [11, 13, 15, 16, 19]. Figure 1 describes this flow and return of data output from EMS, MCAs, MDHHS, and NEMSIS. Analysis of NEMSIS, and MI-EMSIS, variables can inform performance assessments aimed at improving the quality of prehospital care [17].

### Importance

Prior studies suggest that EMS oversight requires not only involvement in quality improvement (QI) through medical direction but also through creating quality monitoring and improvement infrastructure such as statewide QI programs [18]. This is best done through comprehensive datasets and consistent reporting from local EMS agencies to oversight authorities [18, 20]. The most basic measure of dataset quality is completeness, (i.e., lack of invalid values or missing data) [1]. In its 2015 report, NAM stated that an absence of a data registry that captured high-quality and complete demographic data regarding race/ethnicity and other socioeconomic factors made it challenging to gather and evaluate evidence on disparities in cardiac arrest incidence, treatment, and outcomes in the prehospital setting [1].

An analysis of completeness has been previously conducted on 2008–2012 NEMSIS to observe trends in missing and invalid values [21]. It found that data elements traditionally found on EMS patient care reports with finite code sets were most often non-missing and valid (e.g., patient gender). However, current categorical elements attempting to characterize patient information that were previously documented as narrative (e.g., provider’s impression of the patient’s condition) were more often missing or invalid, displaying up to 60% use of null values. More recent and more in-depth analyses of missingness in this dataset have not been conducted.

### Goals of this investigation

This mixed methods investigation aims to identify areas of data reporting requiring improvement by EMS agencies and oversight entities to MI-EMSIS. What distinguishes this analysis of MI-EMSIS from previous analyses of NEMSIS, aside from being state-specific, was our analysis of EMS agencies grouped at the level of Medical Control Authorities (MCAs).

## Methods

### Quantitative study design and protocol

We retrieved MI-EMSIS data consistent with NEMSIS version 2.2.1 for the years 2010–2015. MI-EMSIS is the statewide Michigan-specific EMS data repository which contributes to the larger NEMSIS. Eighteen variables were chosen based on their clinical significance, relevance to public health, and importance for evaluating EMS quality. Table 1 lists and describes each variable, its type, and criteria for being designated as missing and/or invalid. Briefly, missingness was generally defined as blank values, any data entered using inadmissible values (e.g., zip codes containing letters or fewer than 5 digits; negative values for age, etc.), or any responses recorded as “N/A” when not applicable/available was not a sensible response.

**Table 1:** MI-EMSIS analysis variables. Eighteen variables were chosen based on their clinical significance, relevance to public health, and importance for evaluating EMS quality. The table below displays their types, descriptions, and criteria for being determined missing and/or invalid.

Variable type	Variable	Description	Missing and/or invalid criteria
**Demographics**	Age	Age of patient	Blank or value less than 0 or greater than 115
	Gender	Gender of patient	Blank or not applicable, not available, not recorded, not reported
	Race	Race/ethnicity of patient	Blank or not applicable or not available or not recorded or not reported
**Location**	Patient’s Home Zip	Zip code of patient residence	Blank or more or less than 5 digits, or any alphanumeric characters
	Incident Zip Code	Zip code of incident location	Blank or more or less than 5 digits, or any alphanumeric characters
	Destination Name	Name of patient’s destination facility	Blank
	Destination Code	Type of destination	Blank
**Clinical Narrative**	Chief Complaint Narrative	Provider’s narrative of patient’s chief complaint	Blank
	Provider Primary Impression	Provider’s primary impression narrative	Blank
	Medication Allergies	Patient’s reported medication allergies	Blank
	Medical Surgical History	Patient’s medical/surgical history	Blank
	Current Medication Name	Patient’s current medications	Blank
**Vital Signs**	SBP	Patient’s systolic blood pressure	Blank or alphanumeric or anything other than two or three digits
	DBP	Patient’s diastolic blood pressure	Blank or alphanumeric or anything other two or three digits
	Pulse Rate	Patient’s pulse rate	Blank or alphanumeric or, greater than 300, or less than 5
	Pulse Oximetry	Patient’s pulse oximetry	Blank or negative or less than two digits or greater than 100 or with decimal points
	Respiratory Rate	Patient’s respiratory rate	Blank or more than two digits or alphanumeric characters are invalid
	Body Temperature	Patient’s body temperature	Blank is missing, negative is invalid, less than two digits is invalid

### Quantitative study setting and population

Michigan EMS agencies were grouped by their respective MCAs, which were identified using an MCA-Agency Directory provided by MDHHS.

### Quantitative key outcome measures and analysis

The proportion of missing or invalid values for these 18 variables were assessed over the years 2010–2015, overall and stratified by EMS agency, software platform, and MCA. Analysis by MCA required the creation of a new MCA variable. This variable was created by grouping EMS Agency IDs by their respective MCAs, which were identified using an MCA-Agency Directory provided by MDHHS. In 2015, 28 software platforms were used for data entry but just six of them accounted for 82% of the data entered (Figure 6 of the Appendix); these six were chosen for analysis by software. Analyses of the levels of variable missingness were performed in SAS (Statistical Analysis Software) [22].

The quantitative analysis was conducted at the incident level and included *all* recorded incidences recorded. In 2015, approximately 9% of calls were either cancelled, had no patient found, required no treatment, or made no patient contact.

### Qualitative

#### Qualitative study design and protocol

Using the results from a systematic review of the literature on EMS quality measurement [17] and an analysis of MI-EMSIS, we developed a guide for focus groups and key informant interviews. We conducted four 90-min focus groups with 5 to 13 participants, and ten 60-min key informant interviews as part of a broader evaluation of quality measurement of EMS oversight in MI. Among the goals of these focus groups and one-on-one interviews was to identify challenges to EMS agencies to establishing more reliable and valid data collection and reporting to MI-EMSIS.

We developed the recruitment strategy for focus group and one-on-one interview participants with input from MDHHS partners. Using contact information from a publically available MCA Directory provided by MDHHS, we contacted potential participants by email and phone. From those expressing interest, final key informant (Table 5 of the Appendix) and focus group participants were selected (Table 6 of the Appendix).

Both focus groups and interviews were conducted to leverage the strengths of each. Interviews allowed for in-depth discussions with MCA directors and QI coordinators, while focus groups allowed for broader, interactive discussions among EMS coordinators, paramedics, as well as directors and QI coordinators.

One of three study members (MA) served as the moderator for each focus group and 10 one-on-one interviews, while another member (RT) took detailed notes. Interview and focus group guides began with questions on factors that affect successful EMS oversight by MCAs, then covered specific areas such as QI efforts. After these topics, participants were asked “How does data collected as part of MI-EMSIS fit into evaluation and QI at your MCA?”

#### Qualitative Study Setting and Population

Participants included Michigan MCA medical directors and executive directors; hospital administrators and QI coordinators; EMS coordinators; paramedics; and emergency medical technicians. Participants were selected purposefully to ensure representation from different MCAs; urban, rural, and suburban settings; professional roles; and the various regions of the state (based on the state’s eight trauma regions). State trauma regions are comprised of local MCAs that coordinate as a network to oversee regional preparedness and provide trauma care oversight [20]. Sampling MCA participants based on trauma regions allowed for the representation of geographically distinct areas as well as providing rapport between focus groups members, and providing a shared understanding of their region’s practices, needs, and relationships, all of which can contribute to reducing barriers to discussion among focus group participants.

#### Qualitative key outcome measures and analysis

We used a rapid analysis technique, a team-based method of ethnographic inquiry using triangulation and iterative data analysis to develop actionable information from an insider’s perspective to inform policy (24,25. We developed a transcript summary template structured by questions from our interview guide. After each interview or focus group, 2 study team members (RT and another analyst) independently extracted responses from the transcript into the structured rapid analysis template in the form of summary statements and illustrative quotes. They then met to create a final summary by consensus. When summaries were complete, RT extracted data from the summaries into a matrix in Microsoft Excel, ordered by transcript and summary template categories [23]. RT summarized common themes from the matrix to report results.

### Mixed methods key outcome measures and analysis

In order to match qualitative themes with quantitative findings related to MI-EMSIS, RT and SH constructed a 2-column matrix that consisted of a column for each quantitative finding and a column for qualitative themes. The items within each column were then rearranged, first so that conceptually similar results within each column were contained within the same cell, and second so that qualitative themes related to quantitative results appeared side-by-side. Then, in a mixed methods analysis, a third column was created for interpretive statements derived through integrating quantitative findings and qualitative themes in each row. This rearrangement and inductive reasoning through a joint display constitutes mixed-methods analysis. A final joint display was created from the described analytical joint display to present synthesized findings (Table 3).

The large amount of nuanced quantitative and qualitative results that descriptive analysis of the MI-EMSIS database and participant input provided, and the need for these results to inform one another’s interpretations, called for the immersive nature of the joint display and a need for visually laying out, handling, integrating, and synthesizing all data in conjunction to reduce common errors in attempting mixed-method analysis such as recall bias or findings that are not truly mixed but simply presented in parallel [24].

## Results

### Quantitative results

#### Incident-level analyses

**Fig. 2:**
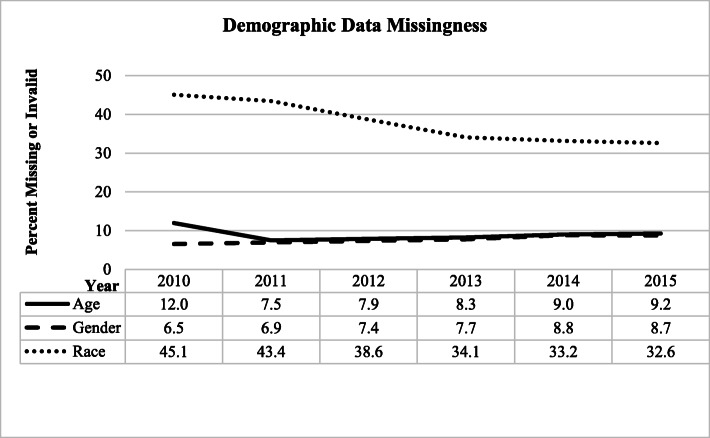
Demographic Data missingness at the incident level

**Fig. 3:**
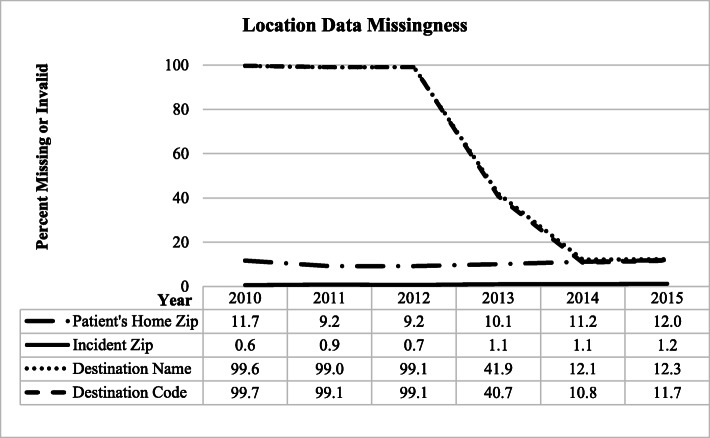
Location Data missingness at the incident level

**Fig. 4:**
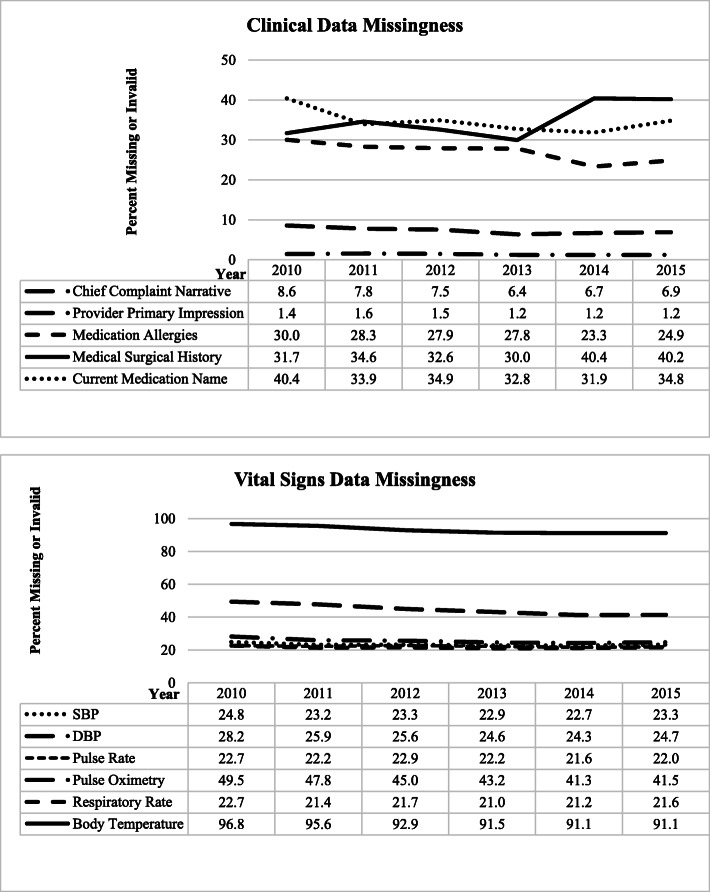
Clinical and Vital Signs missingness at the incident level

Most variables showed consistent trajectories in data reporting over time (Figs. 2, 3, and 4), with little changes in the proportions of missing or invalid values, with the exception of relatively large decreases in the proportion of missing or invalid values for Destination Name and Destination Code.

#### Software-level analyses

**Fig. 6:**
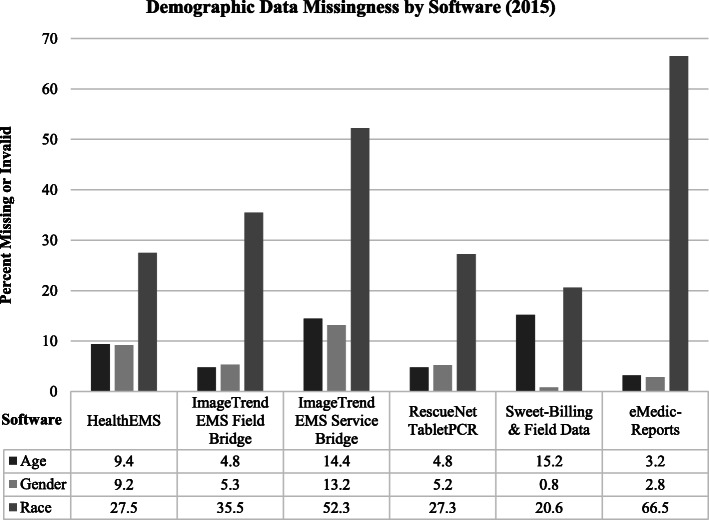
Demographic Data missingness by software for 2015. *Demographic Data missingness:* for demographic variables, eMedicReports displayed the lowest rates of missingness for age, while Sweet-Billing & Field Data showed the lowest rates of missing for gender and race

**Fig. 7:**
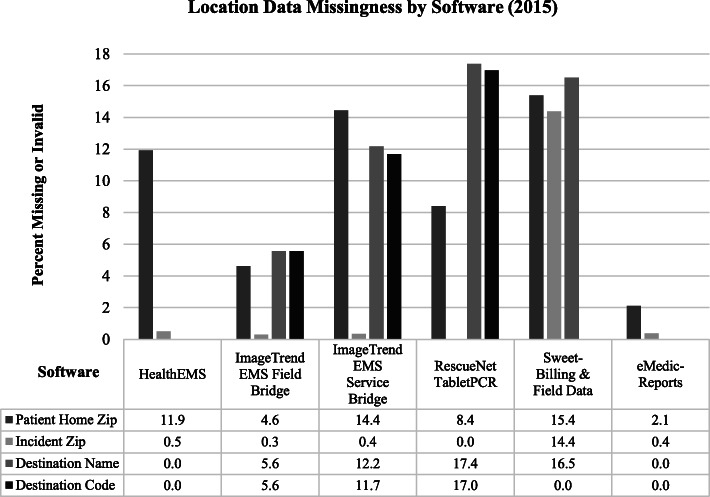
Location Data missingness by software for 2015. *Location Data missingness:* for location data variables, MedicReports exhibited the lowest rates of missing or invalid values for all variables except Incident Zip, which was exhibited by RescueNet TabletPCR

**Fig. 8:**
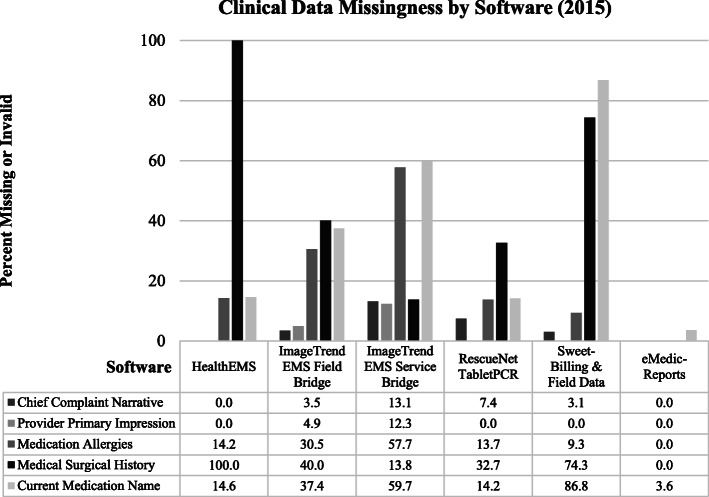
Clinical Data missingness by software for 2015. C*linical Data missingness:* for clinical data variables, eMedicReports exhibited the lowest rate of missing or invalid values for all variables

**Fig. 9:**
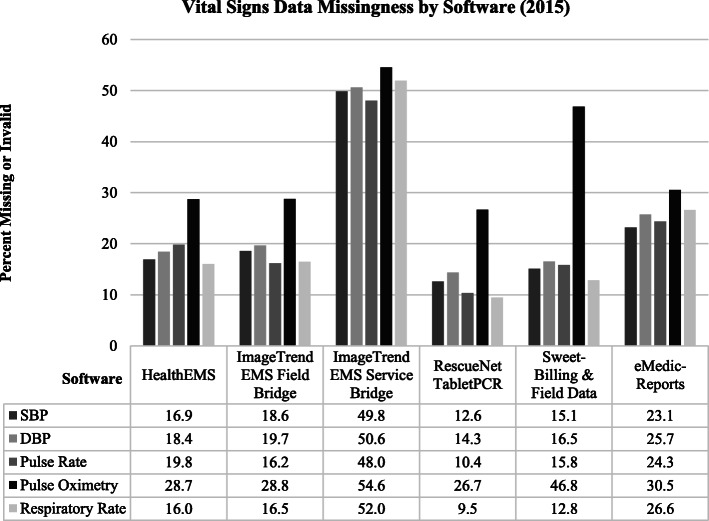
Vital Signs Data missingness by software for 2015. *Vital Signs Data missingness:* for vital signs data, RescueNet TabletPCR exhibited the lowest rates of missing or invalid values for every category of vital signs data

We observed variation in missingness over time and across those platforms accounting for the majority of incidences (Figure 6–9 of the Appendix). For demographic variables, eMedicReports displayed the lowest rates of missingness for age and race, while Sweet-Billing & Field Data showed the lowest rates of missing for gender. For location data variables, eMedicReports exhibited the lowest rates of missing or invalid values for all variables except Incident Zip, where it was outperformed by RescueNet TabletPCR. For clinical data variables, eMedicReports exhibited the lowest rate of missing or invalid values for all variables. For vital signs data, RescueNet TabletPCR exhibited the lowest rates of missing or invalid values for every category of vital signs data.

#### Agency-level analyses

A total of 811 EMS agencies residing in 61 MCAs submitted 8,237,550 EMS activations between 2010 and 2015. Table 2 presents descriptive statistics for the proportion of missing/invalid values at the agency level, showing substantial variation in data completeness for most variables. Those variables with relatively less missing/invalid values overall (i.e., incident zip and provider primary impression) typically show less variation in missingness across agencies, with the exception of Body Temperature, which shows little variation in missingness but over 90% missingness on average.

**Table 2:** Agency-level data missingness descriptive statistics (2015)

	Mean	Median	Standard deviation	Range
**Age**	17.9	6.8	26.4	0–100
**Gender**	17.3	5.8	27.2	0–100
**Race**	18.8	7.3	27.5	0–100
**Patient Home Zip**	18.8	7.3	27.5	0–100
**Incident Zip**	0.8	0	6.9	0–100
**Destination Name**	16.5	4.4	27.8	0–100
**Destination Code**	17.9	4.3	29.7	0–100
**Chief Complaint Narrative**	16.7	2.9	30.3	0–100
**Provider Primary Impression**	6	0	15.4	0–100
**Medication Allergies**	53	50	39.6	0–100
**Medical Surgical History**	27.2	5	38.6	0–100
**Current Medication Name**	67.7	86.2	35.3	0–100
**Systolic Blood Pressure**	54.8	52.9	36.2	0–100
**Diastolic Blood Pressure**	55.8	54.5	35.7	0–100
**Pulse Rate**	52.4	46.3	36.6	0–100
**Pulse Oximetry**	59.4	58.3	34.2	0–100
**Respiratory Rate**	57.3	63.7	37.4	0–100
**Body Temperature**	95.2	100	10.2	17.7–100

#### MCA-level analyses

For 2015 data, there was substantial missing and/or invalid data across MCAs (mean > 10%) for all variables with the exception of Incident Zip Code (1.6%), Chief Complaint Narrative (8.7%), and Provider Primary Impression (3.1%). MCA-level analyses are presented in Table 8 of the Appendix. There was also a large degree of heterogeneity (Standard deviation > 10%) in the missingness levels of all variables with the exception of Incident Zip (8.7%), Destination Name (9.2%), Chief Complaint Narrative (8.4%), and Provider Primary Impression (4.7%).

### Mixed-methods results

Our qualitative findings support and explain the results of our quantitative analyses in 3 areas: MCA- and agency-level variations, data quality, and utility. In each area, we present integrated quantitative and qualitative findings, which are summarized in Table 3. Table 4 presents supporting quotes from qualitative analysis of interviews and focus groups.

**Table 3:** Joint display of findings

Mixed-method themes and subthemes	Quantitative findings	Qualitative findings
*Variation*		
Agency and MCA-level variation	At the agency level, average (mean) rates of missing or invalid values for the year 2015 were consistently larger than missing or invalid values for the same variables at the incident level. MCA-level results also show significant varying rates of missingness by MCA.	Agency: While some participants reported that they were confident in the completeness and quality of their own agency’s data, others acknowledged that data entry was often a problem for their agencies.
		MCA: Participants reported this in the context of MCAs as well whereby certain MCAs perform better in data collection or have more resources to do so.
Software and data mapping variation	Different software platforms exhibited greater or lesser levels of missingness.	Participants expressed that much of the variation in data completeness and quality was due *to* data mapping issues, which is primarily a result of the data reporting software used.
*Data quality*		
Data quality: data entry	Of the 18 variables studied, only five exhibited less than 10% missingness, while only Incident Zip Code and Provider’s Primary Impression exhibited less than 5% missingness.	Participants expressed frustration that despite the time and effort that is required to collect and report high-quality data, the resulting dataset has levels of missing or invalid data that make them of limited use to QI efforts.
Data quality: “bare-minimum” effect	At the agency level, *required* demographic data was found to be missing or invalid at much lower rates than Vital Sign Data. Age, Gender, and Race were missing or invalid 17.9, 17.5, and 18.8% of the time, respectively. This is notably lower than for unrequired Vital Signs Data such as Medical Allergies (53%), Current Medication Name (67.7%), Pulse Oximetry (59.4%), and Body Temperature (95.2%).	During interviews, key participants referred to the fact that data reporting software is not used to best practice. Instead, they claimed that the bare minimum amount of data is often entered into reports in order to meet reporting and compliance requirements.
*Utility*	There was no MCA level variable for regional oversight. The system itself is difficult to query, requires the downloading of many files and statistical expertise.	Many participants expressed discontent that there was no way to query MI-EMSIS to answer clinically relevant questions and that the lack of regional identifiers (MCA or county variables) made oversight using MI-EMSIS difficult.

**Table 4:** Qualitative themes and illustrative statements

Theme	Illustrative quotes
**Data mapping issues between MI-EMSIS and data entry software**	“We can query it (MI-EMSIS) and when you look at the data you know it’s not valid. It tends to be a data entry or data mapping issue (…) If there was a way to map data correctly it would eliminate that. The ultimate dream would be linking prehospital data to hospital data.” – *Key informant, Medical director, Suburban/Rural MCA*
	“It’s [data completeness] somewhat dependent on the software the agency uses. Some upload seamlessly to the state—some have huge problems.” – *Focus group 2, Medical director, Suburban/Rural MCA*
	“For agencies that use non-image trend software, it’s really difficult to get it to match up.” – *Focus group 2, EMS quality improvement director, Suburban/Rural MCA*
	“My agencies put in good, uncorrupted, relatively complete data. The minute it gets uploaded it’s no longer good or uncorrupted.” – *Focus group 3, Executive director, Suburban/Rural MCA*
	“At least for us I don’t believe it is poor or inadequate data entry. I know for a fact that with my three agencies accuracy is well over 90% and the problem is mapping into ImageTrend. The state system corrupts the data. I don’t know how that happens, but I can go to all my agencies and they can provide me with the exact same raw data and I’m comfortable that it’s 90-95-98% accurate, but at the state-level the same data is at best 40% accurate.” – *Focus group 3, Executive director, Suburban/Rural MCA*
	“Again their tracking reports are good but when it gets connected to MI-EMSIS things fall apart. I think that the goal of following a patient is great but we are nowhere near it.” – *Focus group 3, EMS coordinator/paramedic, Suburban/Rural MCA*
**Resources**	“So a process was put in place (to use MI-EMSIS) because of a federal push but there hasn’t been any investment in making it reasonable and functional. [We have] One data manager for the whole state.” *– Focus group 2, Executive director, Suburban/Rural MCA*
	“Some of the smaller areas don’t have the infrastructure needed like call stations or computers even to enter the information.” *– Focus group 2, EMS coordinator, Suburban/Rural MCA*
	“I am not a data expert. I have struggled so hard to understand this data stuff and how to make ours fit into the state system and I have had practically no one helping me figure this out and I feel like I have no one to go to for the technical assistance needed to do this well. If the state would just give us some meaningful help to understand the data, the data elements and how we get them from one vendor to another in a meaningful way, that would be great.” – *Focus group 2, EMS coordinator/paramedic*, *Suburban/Rural MCA*
	“I have gone through seminars around the state—some very well attended—trying to learn and found myself having wasted my time and getting nothing out of it to help me make the data better going into MI-EMSIS. Until the state gives us leadership on this issue nothing is going to change.” – *Focus group 3, Executive director, Rural/Urban MCA*
	“It (improving quality) requires resources, we can’t do mandatory things unless they get paid, without getting paid, there’s no-way to budget for it. I can’t charge the service; it makes it tough to get info out there. Voluntary education can only go so far. Even with high powered professors teaching, very few people show up because we can’t mandate the education.” *– Focus group 1, Medical director, Urban/Suburban MCA*
**Unclearly defined variables**	“[Variable] definitions aren’t consistent…What is an [intubation] attempt? Some of this is actually a national issue and this makes MI-EMSIS unreliable because you never know what is intended at the provider standpoint.” *– Focus group 2, Medical director, Suburban/Rural MCA*
	“You quickly find that the info. is there, but the way it’s labeled makes it impossible to pull out clinically relevant info. So we just go to the agencies and the hospitals and collate those ourselves. It works but it’s labor intensive.” – *Key informant, Medical director, Suburban/Rural MCA*
	“There is 50-60% inaccuracy levels on data reports. We can’t get data driven reports with this data.” – *Focus group 3, EMS coordinator, Rural/Suburban MCA*
	“Unless there is some guide with definitions that reads to all people the same way, like “an intubation attempt means…” This will make everyone across the state start reporting the same. But how the heck do you do that across the state with so many providers. I don’t know that—but that’s the first step. Making sure everyone know what is meant by reporting in each field.” *– Focus group 2, Medical director, Suburban/Rural MCA*
**Low-quality data entry & a need for training**	“We do not—the vast majority do not—utilize their reporting system to its best practice, but instead, to the bare minimum to get the report done. And that’s one reason I don’t like MI-EMSIS.” *– Focus group 4, EMS coordinator/paramedic, Rural MCA*
	“There’s just a misconception that if there’s some data that’s in a computer it’s always right. But up here [in rural areas] where we have a lot of people who do a handful of runs a year, you are going to get a lot of poor data entry because the systems are complex. It should be simplified to what is really necessary and quick to input.” *– Focus group 4, Medical director, Rural MCA*
	“It [MI-EMSIS] is not user friendly or possible for someone untrained to databases, etc.” *– Focus group 2, Quality improvement director, Suburban/Rural MCA*
	“One of the key issues is people don’t know how to use the software properly and are not trained properly. They aren’t trained to use MI-EMSIS or whatever vendor they are using so we are getting poor data.” – *Focus group 4, EMS coordinator/paramedic, Rural MCA*
	“There’s this system that the state spends so much time and money on MI-EMSIS but it’s of no use because it’s so non-functional. If the state could really make this work it would be a huge tool.” – *Focus group 2, Executive director, Suburban/Rural MCA*
**Utility of MI-EMSIS**	“MI-EMSIS right now has zero relevance to an MCA. There is zero capacity in the vendor software to run an MCA level report so I go to each of the three that work in my MCA, we collate data and then I look at that.” – *Focus group 3, Executive director, Rural/Suburban MCA*
	“The goal, as I understood, was to follow a patient from MFR [Medical First Response] to outcome and none of that is linked.” – *Focus group 3, EMS coordinator, Rural/Suburban MCA*

#### Agency- and MCA-level variations

At the agency level, average (mean) rates of missing or invalid values for the year 2015 were consistently larger than missing or invalid values for the same variables at the incident level. This means that between agencies, there are more differences in missing and/or invalid values than between incidences.

While some interview and focus group participants reported that they were confident in the completeness and quality of their own agency’s data, others acknowledged that data entry was often a problem for their agencies.

MCA-level quantitative results show varying rates of missingness by MCA with particular MCAs exhibiting significantly lower rates of data completeness.

### Resources

Participants reported that differences in resources available to MCAs may lead to MCA-level variation. The ability of MCA personnel to commit time, effort, and focus to improve data quality depended on the availability of resources in each MCA’s region. For example, certain MCAs perform better in data collection because they have the necessary staff, infrastructure, and training. Further, participants from the more resource-deprived MCAs reported having more difficulty generating the funds to improve their contribution to MI-EMSIS. MI-EMSIS reporting, including data entry and uploading data, is time-consuming and requires proper infrastructure. Many participants reported that lack of infrastructure—such as computer technologies and the personnel to maintain them—contributed to difficulties such as determining why certain software platforms upload seamlessly to MI-EMSIS and others do not.

### Software and data mapping issues between data entry software and MI-EMSIS

Our quantitative findings show observable differences in levels of missing and/or invalid values by software type. Whether or not that is directly due to the software or is correlated with which agencies use that particular software is uncertain. Our findings do show significant variation by agency as well.

In interviews and focus groups, participants reported that differences in the functionality and availability of data fields were contingent on the choice of software used by EMS providers for data entry to MI-EMSIS. Further, they stated that much of the variation in data completeness and quality stemmed from data mapping issues (i.e., technical incompatibility between the data entry software EMS agencies use during or after incidences and the MI-EMSIS data repository). Uploading agency data to the state dataset resulted in lost or corrupted data. While some participants advocated reducing this variation in software across EMS agencies, some indicated that the EMS agency should determine platform choice, not the state or MCAs.

### Data quality

Our quantitative analyses found that MI-EMSIS data is of low quality; of the 18 variables studied at the incident level, only five exhibited less than 10% missingness, only Incident Zip Code and Provider’s Primary Impression exhibited less than 5% missingness.

Participants expressed frustration that despite the time and effort that is required to collect and report high-quality data, the resulting dataset has levels of missing or invalid data that make them of limited use to QI efforts.

### Unclearly defined variables

Our qualitative findings suggest that unclearly defined variables is a large contributor to low-quality MI-EMSIS data. Most participants reported that although uniform definition of MI-EMSIS variables is required for oversight agencies to conduct high-quality oversight, many variables were unclearly defined. To address this problem, participants suggested that MDHHS provide a dictionary of terms and concepts to support correct and consistent data entry.

### Low-quality data entry and the “Bare Minimum” effect

Quantitative analyses showing that the types of variables found to be least missing and/or invalid and the qualitative themes suggesting low-quality or minimum data entry suggest a “Bare Minimum Effect” whereby EMS field-staff may be stopping data entry at the point where the report is deliverable but no further. Indeed, we found from our quantitative analyses that there is less heterogeneity in missing and/or invalid values for those variables that are required data elements. At the agency level, required Demographic data was found to be missing or invalid at much lower rates than Clinical or Vital Sign Data. Age, Gender, and Race were missing or invalid 17.9, 17.5, and 18.8% of the time, respectively. This is notably lower than Medical Allergies (53%), Current Medication Name (67.7%), Pulse Oximetry (59.4%), and Body Temperature (95.2%).

During interviews, key participants also suggested that data reporting software is not used to best practice. Instead, they claimed that the “bare minimum” amount of data is often entered into reports in order to meet reporting and compliance requirements.

Many participants cited low-quality data entry as a key barrier to using MI-EMSIS effectively. Some participants perceived this as resulting from EMS personnel doing the “bare minimum” to comply with requirements.

### Low-quality data entry and a need for training

Some participants attributed data entry issues to a lack of training on using the software. Making the software easier to use was also suggested as an alternative to increasing training.

### Utility of MI-EMSIS

The previous theme of data quality is related to but does not encompass all utility issues. Our study team found no MCA-level variable for regional oversight and needed to create one for our analyses using agency IDs. The system itself is difficult to query and requires the downloading of many files and statistical expertise that may not be available to MCAs.

Many interview and focus group participants stated that MI-EMSIS was not clinically relevant to them and did not support oversight because it lacked MCA-level variables. This left no capacity to run regional-level reports, nor to query MI-EMSIS to answer clinically relevant questions.

Further, they suggested a pattern of disuse and dysfunction in the MI-EMSIS dataset, partly because there is “no teeth” to the MDHHS requirement to comply with MI-EMSIS data input. Nonetheless, they felt that MDHHS should guide software compatibility improvement or education on data entry.

## Discussion

Precise measurement of patient outcome variables that account for sources of potential confounding or bias is essential for advancing the current understanding of the quality of prehospital care delivery and to guide future QI initiatives in prehospital care. Of the 18 variables included in this analysis, only five exhibited less than 10% missingness in the year 2015: Chief Complaint Narrative, Providers Primary Impression, Incident Zip Code, Gender, and Age, while only Incident Zip Code and Provider’s Primary Impression exhibited less than 5% missingness. Analyses with this level of missingness would likely not pass peer review and would in all likelihood be treated cautiously by QI professionals.

At the agency level, we noticed that average (mean) rates of missing or invalid values by agency for the year 2015 were consistently larger than missing or invalid values for the same variables at the incident level, suggesting there are agencies with very high levels of missingness that are raising the average rate of missingness. Supporting this is the observation that for all but three of the variables studied at the agency level (Current Medication Name, Respiratory Rate, and Body Temperature), the mean was higher than the median. Their difference indicates that agencies with high rates of missingness are having a strong effect on the mean, despite representing fewer than half of the agencies in the study. Relatedly, standard deviations for missingness are very large—around 15 to 37%—and ranges were wide for most variables studied. This wide variability indicates that improving the worst-performing districts could drastically improve the overall average. Furthermore, this points to large between-agency differences in the barriers to accurate reporting. MI-EMSIS, like other datasets, faces challenges related to data entry and reporting originating from the contributing EMS agencies. If agencies have different definitions or internal protocols for data entry including direct entry into the system or data entry into third-party software for importation into the system and what personnel are responsible for data entry (i.e., medical versus non-medical personnel), comparative analyses of these data may not be appropriate. For example, all levels of providers write reports, and thus perform data entry. Also, in some systems, non-healthcare personnel enter the information from paper runforms that were written by the provider (Kevin Putnam, State EMS Data Manager). For the version analyzed (NEMSIS v2.2.1.), the only true validation for importing services is the NEMSIS XSD that the file must pass. For those entering directly into the system, there are validation rules that will deduct from the validation score and flag warnings in the form, but it does not force the user to adhere to the rules (Kevin Putnam, EMS State Data Manager).

The proportion of missing data is directly related to the quality of statistical inferences. This means that datasets with large amounts of missingness may be unusable. Although there are no formally established cutoffs in the literature regarding acceptable proportions of missing data, there are assertions that analyses would be biased when missingness is greater than 10% and largely inconsequential if less than 5% [25, 26]. Several different approaches are taken to address missingness in large datasets. Techniques referred to as single or multiple imputation attempt to “mimic” missing data by providing an “informed guess” at a missing data point by substituting the blank value with a mean value, or the results of a regression equation or even multiple simulations of other observed values [27]. Multiple imputation and other more simplistic approaches such as list wise deletion and mean imputation rely on the data being “missing at random”, a technical condition meaning that, conditional on all measurable variables, missingness is independent on the value of the missing variable. Missing at random cannot be proved statistically [28] but must be reasoned substantively; this condition may be reasonable in the circumstances described here, but having the veracity of inferences rely on an uncheckable condition should be avoided if at all possible.

In the year 2015, 28 software platforms were used but just six platforms accounted for 82% of this data. Of these six, incidents reported through the platform eMedicReports exhibited the lowest levels of missing/or invalid data for most but not all variables. Assuming the agencies using this platform are not substantially different from those using other platforms, we may assume that eMedicReports is more usable and/or has fewer compatibility issues with MI-EMSIS. Given variation in missingness by software platforms, we believe there may be different extents of data mapping issues between different software platforms and MI-EMSIS. This is supported by statements from focus group participants, who reported that much of the variation in data completeness and quality was due to software-specific data mapping issues. However, the quantitative data indicate that data mapping may not explain all of the data imperfections. There are always software vendor changes which occur, and it is difficult to say whether this had any impact on data collection for the time period covered by this project (Kevin Putnam, EMS State Data Manager). Additionally, the data obtained for the study was all based on NEMSIS v2.2.1, so NEMSIS criteria changes are not a factor in the results. At the agency level, Patient Home Zip was found to be missing or invalid 18.8%, while incident was found to be missing or invalid only 0.8% of the time. If certain data types do not map well, one may expect to see similar rates of missingness across similar data types, such as zip codes. The fact that Patient Home Zip Code and Incident Zip Code have different levels of missingness may indicate that data mapping does not explain all of the imperfections in the data, suggesting that incomplete data entry and other factors may play a role as well.

Participants indicated that data entry is affected when the staff members who enter the data only enter the bare minimum necessary to comply with reporting requirements. Our quantitative findings corroborate this phenomenon. At the agency level, Demographic data was found to be missing or invalid at lower rates than both Clinical and Vital Sign Data. Age, Gender, and Race—which are required data elements [29]—were missing or invalid 17.9, 17.5, and 18.8% of the time, respectively. This is far lower than for Clinical or Vital Sign Data such as Medical Allergies (53%), Current Medication Name (67.7%), and other non-required data elements.

The fact that vital signs are missing in many encounters’ needs is notable as vital signs for transported patients are generally recorded. The observed missingness in our study may suggest that missingness might be clustered around patients that are not transported or from agencies that are first responders and hand over the patient to a transporting service, who subsequently provides and documents vital signs. This points to a need for identifying unique patients among EMS activations to be able to conduct more assessment of missingness and more accurate analyses using these data.

Because MI-EMSIS does not contain a variable denoting which MCA data originates from, the analysis of missingness at the level of individual oversight entities was not readily doable, which points to a simple avenue for future data quality improvement. Specifically, the study team needed to create a new variable that coded for MCA using a list provided by MDHHS of EMS agencies and their corresponding MCA. Incorporating such a variable for all incidents in MI-EMSIS will make the dataset more usable for future QI efforts, providing both state and local authorities information to guide EMS oversight as MCA agencies are ultimately responsible for MI-EMSIS reporting. Given that there appear to be some MCAs that significantly underreport certain variables, investing in the capability to analyze MI-EMSIS data at the local or regional level will allow evaluation of missingness to determine if it is the result of software usability, data mapping, or data entry issues. Indeed, we found differences by MCA in data missingness, and further investigating this can facilitate regionalization of prehospital care QI, a practice recommended by the NHTSA and others [8, 17, 18].

Finally, forty-seven states currently report data to NEMSIS. According to the 2016 NEMSIS user manual [30], data missingness across localities is variable and in some instances significant. Further, the data missingness is often not at random and, therefore, may bias the results of analyses using related datasets. Hence, although our analysis was focused on MI-EMSIS, our analyses and findings are likely to be pertinent to localities outside of Michigan. Notably, our approach to assessing missingness is generalizable and may be used to assess the completeness of state-level EMS data that is contributed to NEMSIS.

## Limitations

Our study was restricted to a single state. Yet, oversight of EMS performance and, even more so, the use of NEMSIS occurs to some extent in all but two states which are working on reducing barriers to its use; hence, the results and conclusions from this investigation may be applicable outside of Michigan. Further, as noted above, missingness in NEMSIS data has been noted in data reported from other localities across the USA, highlighting the need for conducting such analyses to understand the root causes of and address data missingness.

It is important to note that the incidences in the quantitative analysis at the incident level included *all* incidences recorded. Given that in 2015, approximately 9% of calls were either cancelled, had no patient found, required no treatment, or made no patient contact; (Table 8 of the Appendix) these may be incidences with rightfully missing variables. Lastly, this investigation was conducted as part of a larger statewide investigation relating to quality measurement of EMS systems and oversight. Accordingly, not all of the questions asked during the focus groups and key informant interviews related to data quality or MI-EMSIS. Conducting focus groups and interviews solely on the topic of MI-EMSIS (or NEMSIS) and data quality may have yielded a larger range of findings.

## Conclusions

Quality improvement efforts using outcomes data are an important component of emergency medical services oversight [31]. These and similar analyses should be used to pinpoint areas in data collection and reporting requiring improvement by EMS agencies and their oversight entities in Michigan and other NEMSIS-participating states. Collaboration should occur through state leaders, EMS agency data managers, software developers, and the state MI-EMSIS data manager to develop standard data entry protocols, data validation rules and definitions, and ensure proper data mapping. Such steps are critical in improving the quality of prehospital data so they may be used reliably and effectively in QI efforts and ultimately help improve patient care and outcomes.

## Figures and Tables

**Fig. 5:**
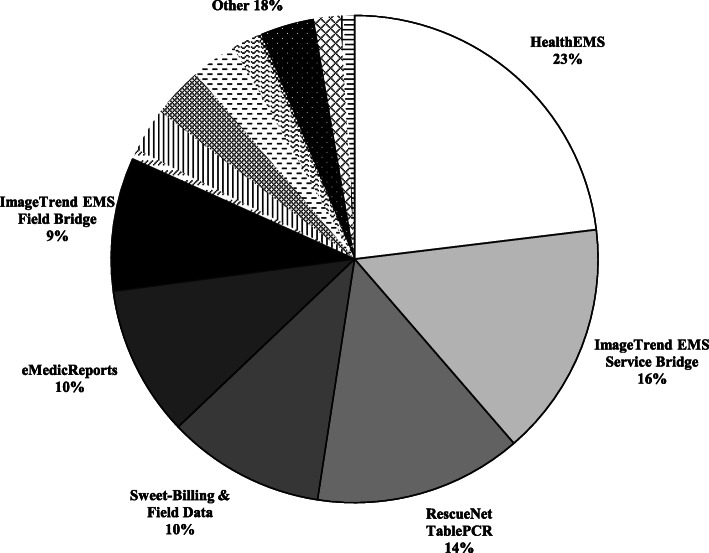
Top 6 most used software platforms in 2015. These platforms account for 82% of the data entered into MI-EMSIS in 2015

## Appendix

**Table 5:** MCA key informant characteristics

Interviewees	Roles	Community setting
1	Medical director	Suburban
2	Executive director	Suburban
3	Medical director	Rural
4	QI coordinator	Rural
5	Executive director	Suburban
6	Executive director	Suburban
7	QI coordinator	Rural
8	QI coordinator	Urban
9	Medical director	Rural
10	Medical director	Urban

**Table 6:** MCA focus group participant characteristics: MI has 8 trauma regions delineated by geographic proximity and similarity as determined by the Michigan Department of Health and Human Services. These trauma regions are responsible for the coordination of trauma care in their areas and were used in sampling MCAs to assure geographic diversity.

Focus groups	Trauma region	Roles	Community setting
1	Regions 2N, 2S and 1	2 Executive directors 1 Medical director 4 EMS coordinators 2 Paramedics 1 QI coordinator	Urban and suburban
2	Region 5	4 Executive directors 5 EMS coordinators 2 QI coordinators 1 Medical directors 1 Paramedic	Suburban and rural
3	Regions 3 and 7	2 Executive directors 2 Paramedics 1 EMS coordinator 1 Medical director 1 QI coordinator	Rural and suburban
4	Region 8	2 Executive directors 1 Medical director 1 EMS coordinator/paramedic 1 EMS coordinator	Rural

**Table 7:** MCA level of missingness analyses

	MCA level of missingness ***N*** (%)							Level of missingness across all MCAs (%)			
	***0–5%***	***5–10%***	***10–15%***	***15–20%***	***20–25%***	***25–30%***	***> 30%***	***Mean***	***Median***	***Standard*** ***deviation***	***Range***
**Age**	13 (21)	24 (39)	7 (11)	5 (8)	4 (7)	0 (0)	8 (13)	13.9	8.4	16.6	0–100
**Gender**	15 (25)	24 (39)	6 (10)	5 (5)	3 (5)	2 (3)	6 (10)	13.7	8	16.5	0–100
**Race**	0 (0)	1 (2)	2 (3)	4 (7)	8 (13)	5 (8)	42 (69)	43.3	39.3	23.4	9–100
**Patient Home Zip**	11 (18)	22 (36)	9 (15)	6 (10)	5 (8)	1 (2)	10 (16)	16.9	9.9	17.8	0–98
**Incident Zip**	58 (95)	1 (2)	1 (2)	0 (0)	0 (0)	0 (0)	1 (2)	1.6	0.1	8.7	0–67
**Destination Name**	20 (33)	13 (21)	9 (15)	11 (18)	5 (8)	1 (2)	3 (5)	11.2	9.5	9.2	0–100
**Destination Code**	24 (39)	12 (20)	6 (10)	11 (18)	3 (5)	1 (2)	6 (10)	12.6	6.4	15.1	0–100
**Chief Complaint Narrative**	24 (39)	24 (39)	4 (7)	3 (5)	2 (3)	2 (3)	2 (3)	8.7	7.2	8.4	0–41
**Provider Primary Impression**	51 (84)	4 (7)	4 (7)	2 (3)	0 (0)	0 (0)	0 (0)	3.1	1.3	4.7	0–20
**Medication Allergies**	2 (3)	3(5)	9 (15)	6 (10)	9 (15)	8 (13)	24 (39)	30.6	27.3	20.3	0–100
**Medical Surgical History**	11 (18)	5 (8)	6 (10)	3 (5)	6 (10)	1 (2)	29 (48)	37.6	24.7	31.3	0–100
**Current Medication Name**	0 (0)	0 (0)	3 (5)	6 (10)	1 (2)	7 (11)	44 (72)	51.7	46.2	27.2	11–100
**Systolic Blood Pressure**	1 (2)	1 (2)	4 (7)	18 (30)	8 (13)	4 (7)	26 (43)	32.5	24.5	21.7	3–100
**Diastolic Blood Pressure**	0 (0)	2 (3)	2 (3)	15 (3)	9 (15)	5 (8)	28 (46)	34.1	25.8	21.4	7–100
**Pulse Rate**	1 (2)	1 (2)	10 (16)	12 (20)	7 (11)	6 (10)	24 (40)	31.2	24.3	21.7	2–100
**Pulse Oximetry**	1 (2)	0 (0)	2 (3)	4 (7)	8 (13)	7 (11)	39 (64)	40.7	35.7	20.8	4–100
**Respiratory Rate**	1 (2)	2 (3)	8 (13)	14 (23)	6 (10)	4 (7)	26 (43)	32.3	24.6	23.1	0–100
**Body Temperature**	0 (0)	0 (0)	0 (0)	0 (0)	0 (0)	0 (0)	61 (100)	90.8	95.4	11.6	44–100

**Table 8:** Incidences with potentially proper missing and/or invalid values

	Year					
Incident’s patient disposition (***N*** %)	2010	2011	2012	2013	2014	2015
**Cancelled**	28,626 (2.9)	47,378 **(**3.7)	56,923 (4.2)	62,400 (4.2)	70,014 (4.5)	71,108 (4.4)
**No patient found**	10,538 (1.1)	14,296 (1.1)	17,194 (1.3)	21,057 (1.4)	23,249 (1.5)	24,123 (1.5)
**No treatment required**	20,833 (2.1)	26,629 **(**2.1)	27,135 (2.0)	28,651 (1.9)	36,789 (2.4)	45,967 (2.9)
**Standby only—no patient contacts**	1993 (0.2)	2555 **(**0.2)	2612 (0.2)	2180 (0.1)	2550 (0.2)	3696 (0.2)
**Total**	61,990 (6.27)	90,858 (7.01)	103,864 (7.64)	114,288 (7.64)	132,602 (8.51)	144,894 (9.04)
